# The Roles of Compensatory Evolution and Constraint in Aminoacyl tRNA Synthetase Evolution

**DOI:** 10.1093/molbev/msv206

**Published:** 2015-09-28

**Authors:** Jeffrey R. Adrion, P. Signe White, Kristi L. Montooth

**Affiliations:** ^1^Department of Biology, Indiana University, Bloomington

**Keywords:** compensatory evolution, gene expression, mitochondrial-nuclear coevolution, protein evolution

## Abstract

Mitochondrial protein translation requires interactions between transfer RNAs encoded by the mitochondrial genome (mt-tRNAs) and mitochondrial aminoacyl tRNA synthetase proteins (mt-aaRS) encoded by the nuclear genome. It has been argued that animal mt-tRNAs have higher deleterious substitution rates relative to their nuclear-encoded counterparts, the cytoplasmic tRNAs (cyt-tRNAs). This dynamic predicts elevated rates of compensatory evolution of mt-aaRS that interact with mt-tRNAs, relative to aaRS that interact with cyt-tRNAs (cyt-aaRS). We find that mt-aaRS do evolve at significantly higher rates (exemplified by higher *d*_N_ and *d*_N_/*d*_S_) relative to cyt-aaRS, across mammals, birds, and *Drosophila*. While this pattern supports a model of compensatory evolution, the level at which a gene is expressed is a more general predictor of protein evolutionary rate. We find that gene expression level explains 10–56% of the variance in aaRS *d*_N_/*d*_S_, and that cyt-aaRS are more highly expressed in addition to having lower *d*_N_/*d*_S_ values relative to mt-aaRS, consistent with more highly expressed genes being more evolutionarily constrained. Furthermore, we find no evidence of positive selection acting on either class of aaRS protein, as would be expected under a model of compensatory evolution. Nevertheless, the signature of faster mt-aaRS evolution persists in mammalian, but not bird or *Drosophila*, lineages after controlling for gene expression, suggesting some additional effect of compensatory evolution for mammalian mt-aaRS. We conclude that gene expression is the strongest factor governing differential amino acid substitution rates in proteins interacting with mitochondrial versus cytoplasmic factors, with important differences in mt-aaRS molecular evolution among taxonomic groups.

## Introduction

Nonrecombining genomes are subject to the accumulation of deleterious mutations due to Muller’s ratchet (Muller 1964; Felsenstein 1974) and linked selection that reduces the efficacy of selection (Hill and Robertson 1966; Charlesworth et al. 1993; Charlesworth 1994; Gillespie 2000). It has been suggested that the effects of linked selection should be compounded in animal mitochondrial genomes, owing to the unique population genetics of mitochondrial DNA (mtDNA) (Gabriel et al. 1993; Lynch and Blanchard 1998; Neiman and Taylor 2009). Relative to nuclear DNA (nDNA), animal mtDNA experiences an elevated mutation rate (Lynch et al. 2006, 2008), does not generally recombine (Ballard 2000; Ingman et al. 2000; but see Piganeau et al. 2004; Gantenbein et al. 2005), and is predominantly maternally inherited, subjecting it to the indirect effects of cytoplasmic elements, such as *Wolbachia*, that can sweep through populations (e.g., Shoemaker et al. 2004)*.* These unique dynamics have led to a well accepted model of mitochondrial-nuclear compensatory evolution, whereby mildly deleterious mitochondrial substitutions are compensated by the fixation of nDNA mutations that restore function (Lynch and Blanchard 1998; Rand et al. 2004; Meiklejohn et al. 2007; Dowling et al. 2008; Oliveira et al. 2008; Osada and Akashi 2012; Barreto and Burton 2013; Sloan et al. 2014).

This model of compensatory evolution predicts that the ratio of nonsynonymous substitutions per nonsynonymous site to synonymous substitutions per synonymous site (*d*_N_/*d*_S_) should be elevated in proteins that interact with mitochondrial-encoded versus nuclear-encoded factors, and that compensatory nuclear mutations should leave a signature of adaptive fixation. Studies of the mitochondrial ribosome in arthropods (Barreto and Burton 2013) and plants (Sloan et al. 2014) support this model, with ribosomal proteins targeted to the organelles having a higher *d*_N_/*d*_S_ than ribosomal proteins targeted to the cytosol. While these findings are consistent with a model of compensatory evolution, a lower degree of functional constraint for organellar, relative to cytoplasmic, ribosomal function is also predicted to result in higher evolutionary rates of organellar-targeted proteins (Sloan et al. 2014). Highly expressed genes have greater amino acid conservation (Pal et al. 2001; Drummond et al. 2005; Drummond and Wilke 2008; Nabholz et al. 2013), and it is hypothesized that this is because highly expressed proteins should be more highly constrained to both fold appropriately (Drummond et al. 2005; Drummond and Wilke 2008; Park et al. 2013) and avoid toxic misinteractions with other cellular components (Yang et al. 2012). This relationship introduces the possibility that the observed differences in *d*_N_/*d*_S_ between organellar- and cytosolic-targeted proteins with similar function may be the result of differential constraint via their respective levels of gene expression.

**Fig. 1.**
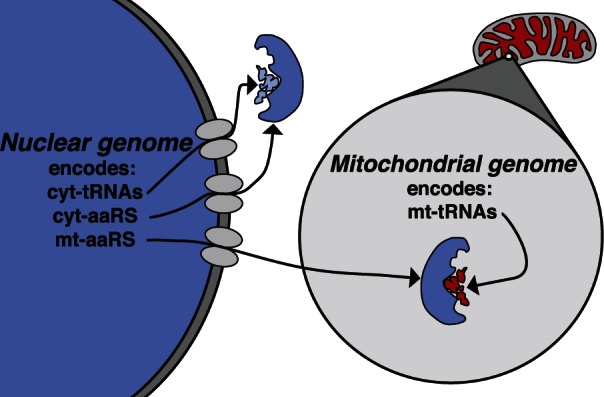
Protein translation requires physical interactions between aaRS proteins and tRNAs. The nuclear genome encodes aaRS targeted to the cytosol (cyt-aaRS) that interact with the nuclear-encoded tRNAs (cyt-tRNAs) and aaRS targeted to the mitochondria (mt-aaRS) that interact with mitochondrial-encoded tRNAs (mt-tRNAs).

The mitochondrial aminoacyl tRNA synthetases (mt-aaRS) that recognize and catalyze the attachment of amino acids onto their cognate mitochondrial-encoded transfer RNAs (mt-tRNAs) are an important class of nuclear-encoded proteins that interact with mitochondrial gene products (Bonnefond et al. 2005; Burton and Barreto 2012; Meiklejohn et al. 2013). Nuclear genomes encode separate classes of aaRS proteins—the mt-aaRS and the cytoplasmic aaRS (cyt-aaRS) that interact with the nuclear-encoded cytosolic tRNAs (cyt-tRNAs) (fig. 1). The ratio of mt-tRNA to cyt-tRNA nucleotide substitution rates varies considerably among taxonomic groups, reaching as much as 25-fold higher in mammals, roughly 9-fold higher in birds, and 4-fold higher in invertebrates, and it has been argued that mt-tRNA substitutions are mildly deleterious due to their inferred effects on tRNA stability (Lynch 1996). Variation among taxa in the ratio of mt- to cyt-tRNA substitution rates further predicts that the signature of compensatory evolution should vary among taxa, with elevated ratios of mt- to cyt-tRNA nucleotide substitution rates driving greater disparity between nuclear-encoded mt-aaRS and cyt-aaRS *d*_N_/*d*_S_.

**Fig. 2.**
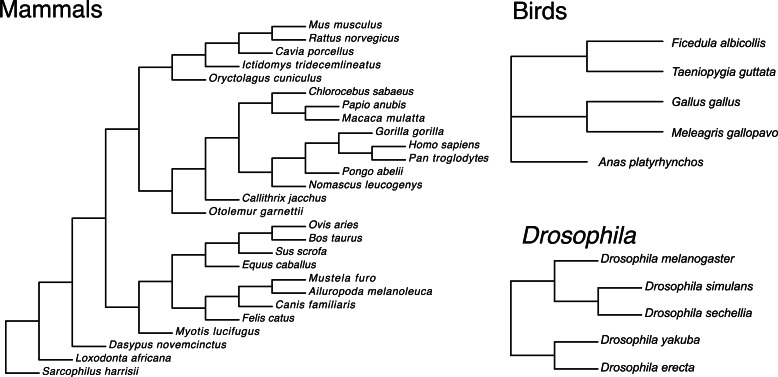
Phylogenetic relationships among species sampled in our analyses. Gene trees, based on gene trees inferred from RAxML and used for the estimation of *d*_N_/*d*_S_ (*ω*) in PAML, rarely conflicted with the known species tree for birds and flies. However, all mammalian gene trees were discordant. The mammalian tree shown represents the known species tree for the full set of mammals used in our analysis (Bininda-Emonds et al. 2007). For some aaRS genes, only subsets of these species were included, based on availability, quality, and length of sequence data.

Here, we quantify patterns of aaRS molecular evolution across 27 species of mammals, five species of birds, and five species of Drosophilid fruit flies (fig. 2) that span a broad range of mtDNA:nuclear substitution rates. We relate levels of aaRS gene expression to protein substitution rates in *Mus musculus* (mouse), *Gallus gallus* (chicken), and *Drosophila melanogaster*, and employ tests of selection using polymorphism and divergence data to investigate the relative contributions of compensatory evolution and constraint to aaRS evolution.

## Results

### mt-aaRS Evolve More Rapidly Than cyt-aaRS

**Fig. 3.**
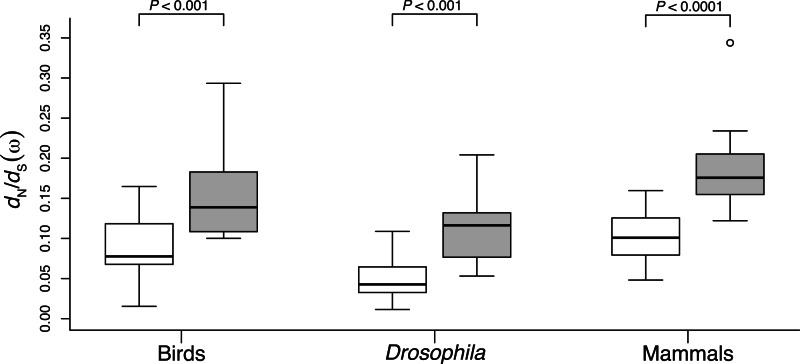
Estimates of *d*_N_/*d*_S_ (*ω*) for nuclear-encoded mt-aaRS (gray boxes) and cyt-aaRS (white boxes). mt-aaRS evolve more rapidly than cyt-aaRS in all taxa analyzed. Estimates of *ω* were generated using codeml (model = 0, NSsites = 0) in PAML. *P* values indicate significant differences based on Mann–Whitney *U* tests.

We estimated *d*_N_/*d*_S_ (*ω*) for 24–31 aaRS genes (depending on sequence availability) using codeml model 0 in the software package PAML v.4.4 (Yang 2007) (table 1). mt-aaRS had significantly greater *d*_N_/*d*_S_ than did cyt-aaRS across bird, *Drosophila*, and mammal lineages (*P* < 0.001 for all comparisons; Mann–Whitney *U* tests) (fig. 3 and table 1), consistent with predictions from a model of mitochondrial-nuclear compensatory evolution. mt-aaRS also had significantly greater nonsynonymous substitution rates (*d*_N_) than did cyt-aaRS across all taxonomic groups (*P*_MWU_ < 0.05 for all comparisons; supplementary fig. S1 and table S1, Supplementary Material online). Synonymous substitution rates (*d*_S_) were not significantly different between mt-aaRS and cyt-aaRS for birds (*P*_MWU_ = 0.80) and mammals (*P*_MWU_ = 0.27). In *Drosophila*, synonymous substitution rates were slightly greater for mt-aaRS than for cyt-aaRS (*P*_MWU_ = 0.04) (supplementary fig. S2 and table S1, Supplementary Material online). *d*_N_/*d*_S_ was not significantly correlated between cyt-aaRS and mt-aaRS that catalyze aminoacylation with the same amino acid (*P > *0.17 for all phylogenetic groups; Pearson’s product-moment correlation), indicating that constraint via this shared function is not driving parallel patterns of molecular evolution in these two classes of proteins.

**Table 1.** Nuclear-Encoded aaRS Sequence Divergence across Taxonomic Groups.

Taxa	aaRS Class	Genes	*d*_N_^a^	*d*_S_^a^	*ω* (*d*_N_/*d*_S_)^b^
Birds	Cytosolic	14	0.019 ± 0.005	0.318 ± 0.123	0.089 ± 0.012
	Mitochondrial	14	0.021 ± 0.003	0.152 ± 0.030	0.151 ± 0.014
*Drosophila*	Cytosolic	12	0.004 ± 0.006	0.070 ± 0.004	0.051 ± 0.009
	Mitochondrial	12	0.011 ± 0.003	0.108 ± 0.037	0.110 ± 0.012
Mammals	Cytosolic	16	0.007 ± 0.001	0.073 ± 0.005	0.104 ± 0.009
	Mitochondrial	15	0.014 ± 0.001	0.077 ± 0.004	0.188 ± 0.014

^a^Per-gene estimates of the average substitution rate per branch ± standard error.

^b^Means ± standard error.

### No Evidence for Positive Selection on aaRS

Mutations that arise in nuclear-encoded mt-aaRS and compensate for fitness loss associated with mt-tRNA mutations should be fixed by recurrent positive selection. Using whole-protein *d*_N_/*d*_S_ to infer adaptive evolution is highly conservative, as the signature of positive selection acting on a relatively small number of sites within functional proteins can be overwhelmed by structural domains under strong purifying selection (Nielsen and Yang 1998; Yang et al. 2000). We estimated *d*_N_/*d*_S_ (*ω*) at individual amino acid sites within aaRS genes by implementing codon evolution models in PAML and calculated the posterior probability that any site was evolving under positive selection using Bayes empirical Bayes (BEB; Yang et al. 2005). Across all aaRS proteins and phylogenetic trees, the positive selection model, M2a, was never a better fit than M1a, nor was there evidence that any amino acid site was evolving under positive selection (*P* > 0.05 for all comparisons; BEB analysis).

### Constraint via Purifying Selection Largely Shapes aaRS Polymorphism and Divergence

To further characterize selection on aaRS proteins, we obtained counts of nonsynonymous and synonymous polymorphic (*P_n_*, *P_s_*) and fixed (*D_n_*, *D_s_*) sites for *D. melanogaster* and humans using *D. simulans* and chimpanzee as outgroup species, respectively. The ratio of *P_n_*/*P_s_* to *D_n_*/*D_s_* (the neutrality index, NI; Rand and Kann 1996) is expected to equal one under a model of strict neutral evolution (McDonald and Kreitman 1991). No aaRS gene rejected neutrality after accounting for multiple tests by using either a Bonferroni correction (Fisher’s exact test; *α* = 0.05) or False Discovery Rate criterion (Fisher’s exact test; FDR = 5%) (table 2). Using summed counts of *P_n_*, *P_s_*, *D_n_*, and *D_s_* across the McDonald-Kreitman (MK) contingency tables, we found that, as a group, cyt-aaRS deviated from the neutral expectation in both *Drosophila* (*P*_FET_ = 0.02) and humans (*P*_FET_ = 0.02), and that mt-aaRS deviated from a neutral expectation in *Drosophila* (*P*_FET_ = 0.03). However, the direction of these departures and that of all significant MK tests before multiple-test correction was consistent with an excess of nonsynonymous polymorphisms (NI > 1), indicative of purifying selection with segregating mildly deleterious variation (table 2). Estimates of NI_TG_, an unbiased estimator of NI (Stoletzki and Eyre-Walker 2011), from counts across MK contingency tables within each class of aaRS, confirmed these patterns (table 2). Moreover, the distribution of NI was not significantly different between cyt-aaRS and mt-aaRS for either humans or *D. melanogaster* (*P*_MWU_ > 0.73 for both comparisons), consistent with these two classes of aaRS experiencing similar levels of constraint.

**Table 2.** Summaries of Nuclear-Encoded aaRS Nucleotide Polymorphism and Divergence in Flies and Humans.

Taxa	aaRS Class	Genes (sig. MK)^a^	Summed MK^b^	Summed OR^c^	Homogeneity^d^	NI_TG_^e^	95% C.I.
*D. melanogaster*	Cytosolic	9 (0)	*P* = 0.02	1.68	*P* = 0.37	2.37	1.37–3.58
	Mitochondrial	9 (0)	*P* = 0.03	1.60	*P* = 0.35	2.02	1.31–3.37
*Homo sapiens*	Cytosolic	16 (0)	*P* = 0.02	2.10	*P* = 0.96	2.07	1.19–3.34
	Mitochondrial	8 (0)	*P* = 0.20	1.89	*P* = 0.85	1.84	0.79–3.82

^a^Number of genes in analysis, with the number of significant MK tests in parentheses. Data are from Langley et al. (2012) for *D. melanogaster* and from Bustamante et al. (2005) for *H. sapiens.*

^b^Significance of Fisher’s exact test of the two-by-two MK table using counts summed across genes.

^c^Odds ratio (NI) calculated from the summed MK table.

^d^Significance of the test of homogeneity among MK tables for each gene.

^e^Unbiased estimator of NI for the gene set as in Stoletzki and Eyre-Walker (2011) with 95% confidence intervals.

### Gene Expression Is a Strong Predictor of aaRS Substitution Rate

**Fig. 4.**
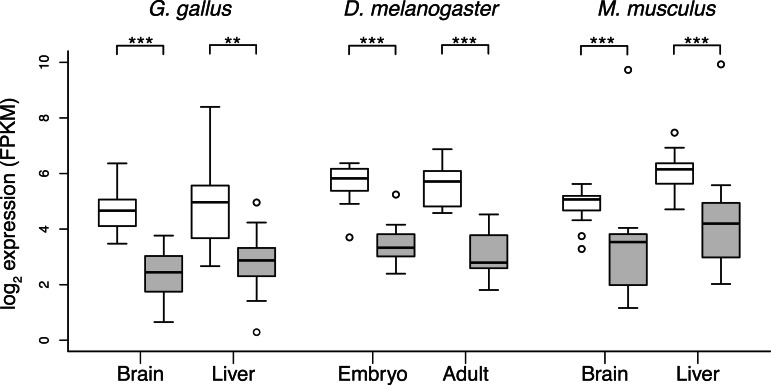
Transcript levels (FPKM) for mt-aaRS (gray boxes) and cyt-aaRS (white boxes). cyt-aaRS are expressed at higher levels than mt-aaRS for all tissues analyzed. Statistical significance: **P < 0.001 and ***P < 0.0001 based on Mann–Whitney *U* tests.

**Fig. 5.**
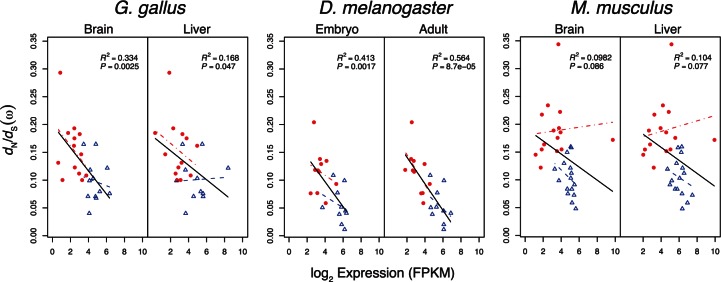
The relationship between transcript level (FPKM) and *d*_N_/*d*_S_ (*ω*) for aaRS genes. A significant negative relationship exists between FPKM and *d*_N_/*d*_S_ in chicken and *D. melanogaster*, but not in mouse. GLM regression lines are shown separately for mt-aaRS (closed circles, red dash-dot line) and cyt-aaRS (open triangles, blue dashed line) and for all aaRS genes (solid black line). *R*^2^ and *P* values are from regressions using data from all aaRS genes.

One of the best predictors of *d*_N_/*d*_S_ for a given gene is the level at which that gene is expressed (Pal et al. 2001; Drummond et al. 2005; Drummond and Wilke 2008; Nabholz et al. 2013). We used available transcriptomic data to characterize the associations between aaRS gene transcript levels and *d*_N_/*d*_S_ for brain and liver tissues in both *M. musculus* (mouse) and *G. gallus* (chicken), and for embryonic and adult life stages in *D. melanogaster*. Expression levels of cyt-aaRS were significantly greater than those of mt-aaRS in all tissues and species considered (*P*_MWU_ < 0.001 for all comparisons; fig. 4), and gene expression explained a significant fraction (17–56%) of the total variance in *d*_N_/*d*_S_ in chicken and flies (*P* < 0.05 for all comparisons; General Linear Model [GLM]) (fig. 5). While there was a trend for transcript levels to be inversely related to *d*_N_/*d*_S_ in both tissues of mammals, the regressions were not significant for these tissues (fig. 5). Gene expression also explained 5–25% of the variance in *d*_N_ (supplementary fig. S3, Supplementary Material online), but less than 6% of the variance in *d*_S_ (supplementary fig. S4, Supplementary Material online), indicating that the relationship between expression and *d*_N_/*d*_S_ is not the result of differences in synonymous substitution rates across classes of nuclear encoded aaRS genes.

### The Relationship between Gene Expression and *d*_N_/*d*_S_ Differs among Taxa

**Fig. 6.**
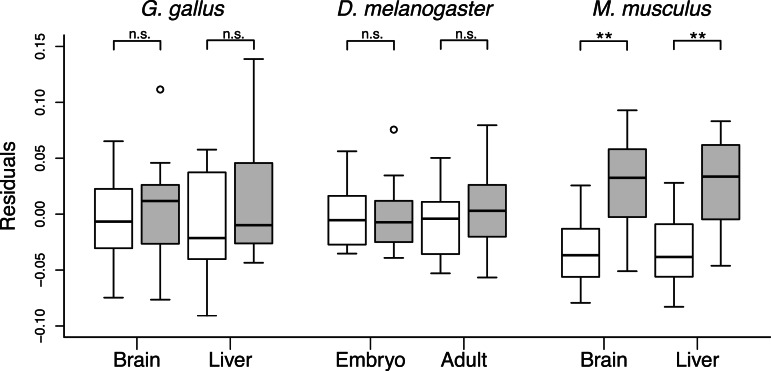
The residuals from GLM regressions of *d*_N_/*d*_S_ (*ω*) on gene expression (FPKM) using data from all aaRS genes for cyt-aaRS (white boxes) and mt-aaRS (gray boxes). In mouse, but not chicken or flies, the pattern of greater *d*_N_/*d*_S_ in mt-aaRS proteins persists after the effects of gene expression are removed. Statistical significance: **P < 0.001 based on Mann–Whitney *U* tests.

A striking pattern is the lack of a relationship between gene expression and *d*_N_/*d*_S_ specifically within the mitochondrial class of aaRS genes in mouse (fig. 5). We used Akaike’s information criterion (AIC) to evaluate and contrast the explanatory power of models that consider: 1) only aaRS protein class, 2) only gene expression, 3) class + expression, and 4) an interaction between class and expression. Both the change in AIC as well as the Akaike weights indicated that for chicken and *D. melanogaster*, models including either expression or class are more equivalent to each other, than is the case for mouse, where the model that excludes aaRS class provides a much worse fit (supplementary table S1, Supplementary Material online). Furthermore, *d*_N_/*d*_S_ no longer differed between aaRS classes in chicken or flies once the variance explained by gene expression was removed (*P*_MWU_ > 0.48 for all comparisons; fig. 6). However, the difference in *d*_N_/*d*_S_ between mt-aaRS and cyt-aaRS in mouse persisted after correcting for gene expression (*P*_MWU_ < 0.001 for both comparisons; fig. 6), indicating that the relationship between aaRS expression and *d*_N_/*d*_S_ differs among taxonomic groups and classes of aaRS genes.

## Discussion

Population genetic theory describing the evolution of nonrecombining genomes (Muller 1964; Hill and Robertson 1966; Charlesworth 1994) laid the foundation for a model of compensatory evolution between the mtDNA and nuclear genomes. Previous studies of molecular coadaptation in mitochondrial-nuclear complexes focused on correlated patterns of amino acid substitution in mitochondrial- and nuclear-encoded OXPHOS proteins (Grossman et al. 2001; Goldberg and Wildman 2003; Osada and Akashi 2012) and have compared *d*_N_/*d*_S_ between nuclear-encoded ribosomal proteins targeted to the mitochondria and the cytosol in particular lineages (Barreto and Burton 2013; Sloan et al. 2014), lending support for a model of mitochondrial-nuclear compensatory evolution. We used a diverse taxonomic sampling of animals and found that aaRS proteins that interact with the putatively more rapidly evolving mt-tRNAs do evolve more rapidly than their cytoplasmic counterparts within each of these taxonomic groups. Although these results are consistent with a model of compensatory evolution, our population genetic and gene expression analyses indicate that a predominant role for compensatory evolution is limited and may explain differential substitution rates of nuclear-encoded proteins that interact with mitochondrial- and cytoplasmic-encoded factors in only a subset of these taxa.

A model of compensatory evolution predicts that compensatory mutations should be recurrently fixed by positive selection to compensate for a persistent influx of deleterious substitutions. Yet, positive selection models were never a better fit to the data than were neutral models, we found no individual amino acid sites evolving under positive selection, and estimates of NI were consistent with a pervasive role of purifying selection. Codon evolution models are best suited for detecting selection on individual amino acid sites that have experienced recurrent selection at the same site (Anisimova et al. 2002). Although this is often an unreasonable assumption, investigation of the crystal structure of a human mt-aaRS suggests that only a small fraction of the total amino acids physically contact the cognate tRNA (Klipcan et al. 2012). Thus, compensatory mutations could potentially be constrained to relatively few sites in aaRS protein, as was the case for nuclear-encoded COX proteins on primate lineages, where positive selection affects seven amino acid sites that putatively interact with the mitochondrial COX proteins (Osada and Akashi 2012). Coupled with inferences of the temporal order of substitutions, this pattern provides convincing evidence for compensatory evolution in COX proteins on particular lineages. Barreto and Burton (2013) also provided evidence for positive selection on two nuclear-encoded subunits of the mitochondrial ribosome in *Nasonia*, with both genes having *d*_N_/*d*_S_ > 1. While Barreto and Burton (2013) found differences in expression level between mitochondrial and cytoplasmic components of the ribosome, they reported no correlation between levels of gene expression and *d*_N_/*d*_S_ within each component, and differences in *d*_N_/*d*_S_ between mitochondrial and cytoplasmic components remained significant after controlling for gene expression level.

In contrast, we found that gene expression explained a significant amount of the variance in *d*_N_/*d*_S_ in aaRS genes with a striking degree of consistency between tissues and life stages in chicken and *D. melanogaster*. Transcript levels were significantly higher for cytoplasmic aaRS relative to mitochondrial aaRS in mouse, chicken, and *D. melanogaster*, presumably because cyt-aaRS and cyt-tRNAs support greater levels of protein translation relative to the mitochondrial compartment. Thus, the molecular evolution of aaRS proteins appears to be largely shaped by constraint via their level of expression, a finding consistent with the idea that differences in constraint between cytoplasmic and mitochondrial protein synthesis can lead to elevated substitution rates in nuclear-encoded components of mitochondrial translation (Sloan et al. 2014; Pett and Lavrov 2015). This difference may be particularly extreme in animal lineages where mt-tRNAs have been lost (Pett and Lavrov 2015; Salinas-Giegé et al. 2015). Yet, there was little relationship between mitochondrial aaRS gene expression and *d*_N_/*d*_S_ in mouse, and the pattern of greater *d*_N_/*d*_S_ in mt-aaRS relative to cyt-aaRS is robust to removing the effects of gene expression in mouse, but not in chicken or *D. melanogaster.* These patterns suggest that the relative roles of constraint versus compensatory evolution may differ in mammals.

The variance in *d*_N_/*d*_S_ that is not explained by gene expression could potentially be explained by variation in copy number and substitution rates of the tRNAs that directly interact with aaRS genes. While the mtDNA encodes 22 tRNAs in all animals used in our analyses, the number of tRNA copies encoded by the nuclear genome varies extensively, from 155 tRNAs in turkey to 4,112 tRNAs in cow (Chan and Lowe 2009). Despite extensive variation in cyt-tRNA copy number among mouse, chicken, and *D. melanogaster*, the relationship between cyt-aaRS gene expression and *d*_N_/*d*_S_ is relatively consistent between these groups (fig. 5). Moreover, variation in mt-tRNA copy number cannot explain the extensive variation in *d*_N_/*d*_S_ of mt-aaRS among taxa, as all but two mt-aaRS genes interact with only a single tRNA copy. Importantly, variation in tRNA substitution rates between genomes could be contributing to the unexplained variance in *d*_N_/*d*_S_ of aaRS genes_._

To test whether variation in mt-tRNA substitution rates is generating elevated *d*_N_/*d*_S_ of mt-aaRS relative to cyt-aaRS genes, we investigated estimates of aaRS *d*_N_/*d*_S_ on branches of our gene trees that had no tRNA substitutions (100% sequence identity between nodes). Under the assumption that compensatory amino acid fixations in nuclear-encoded aaRS occur on the same branch as deleterious mt-tRNA substitutions (but see Osada and Akashi [2012]), we would not expect to see elevated *d*_N_/*d*_S_ of mt-aaRS relative to cyt-aaRS on branches without substitutions in their cognate tRNAs. Across all mt-tRNAs for which we were able to obtain available data, branches without a single mt-tRNA substitution comprise 17% of the total number of branches on the tree in birds, 57% in flies, and 35% in mammals. This high degree of constraint on tRNA sequence evolution casts doubts on a pervasive role for coevolution between mt-tRNA and mt-aaRS. If we estimate aaRS *d*_N_/*d*_S_ for each branch in the phylogeny (using model=1 in PAML) and focus only on branches for which there are no tRNA substitutions with which the cognate aaRS can coevolve, the pattern of significantly higher mt-aaRS *ω* relative to cyt-aaRS persisted across all taxa (*P*_MWU_ < 0.03 for all comparisons). Moreover, in this analysis where mt-aaRS are paired with their cognate mt-tRNA, mt-aaRS *ω* estimates did not differ significantly between branches with tRNA change and branches without tRNA change in any taxa (*P*_MWU_ > 0.24 for all comparisons), strongly suggesting that forces other than compensatory evolution are shaping the evolution of mt-aaRS. However, these analyses are complicated by the fact that model 1 is not always a better fit to the data than model 0, and by the fact that tRNAs experience intramolecular compensatory evolution that can occur within branches (e.g., in *Drosophila* see Montooth et al. 2009), obviating the selection pressure that would favor the fixation of nuclear compensatory mutations.

Why patterns of aaRS evolution differ among taxa remains largely an open question. There are significant differences in effective population sizes (*N_e_*) between mammals, birds, and within the insects (Lynch 2007; Wright and Andolfatto 2008) that may affect the relative contributions of compensatory evolution and constraint via gene expression to aaRS evolution. *d*_N_/*d*_S_ of both mitochondrial- and nuclear-encoded COX proteins scales positively with generation time, a commonly used proxy for population size, in 21 species of mammals (Popadin et al. 2013), and *d*_N_/*d*_S_ of both mitochondrial- and nuclear-encoded OXPHOS proteins in mammals is roughly twice that of *d*_N_/*d*_S_ in birds and insects (Nabholz et al. 2013). Differences in *N_e_* could affect the efficacy of selection for mutations with very small selection coefficients, such as those associated with selection for translational accuracy and efficiency (Akashi 2003; Wagner 2005), both of which underlie proposed mechanisms for the relationship between expression level and substitution rate (Drummond et al. 2005; Drummond and Wilke 2008). A weaker association between gene expression levels and *d*_N_/*d*_S_ in mammals relative to birds and insects is consistent with the reduced efficacy of purifying selection with smaller *N_e_*. However, this cannot easily explain differences between the relationships of *d*_N_/*d*_S_ and mt- versus cyt- aaRS gene expression that we observed within mammals. Importantly, constraint via pleiotropic effects may also be vastly different among taxa, as recent work in humans suggested aaRS splice variants have unique catalytic domains, with biological activities separate from aminoacylation (Lo et al. 2014). These distinct cellular functions could well be contributing to the variance in *d*_N_/*d*_S_ not explained by gene expression.

These patterns lead us to conclude that, while there is little evidence for a predominant role for compensatory evolution in the evolution of aaRS genes, the relative contributions of compensatory evolution and constraint to patterns of aaRS evolution likely vary across animal taxa. Why compensatory evolution would significantly shape the evolution of OXPHOS proteins and mitochondrial components of the ribosome (Osada and Akashi 2012; Barreto and Burton 2013; Sloan et al. 2014), but not that of the mt-aaRS proteins remains an open question that warrants a broader phylogenetic testing for these patterns. Particularly powerful to include are contrasts between lineages that have elevated mtDNA:nuclear substitution rates relative to their closely related species (e.g., Sloan et al. 2014). There is recent evidence that purifying selection is largely effective in the mitochondrial genomes of flies and humans (Cooper et al. 2015), possibly due to greater deleterious selective effects of nonsynonymous mutations that arise in the mtDNA, relative to those arising in nDNA (Popadin et al. 2013). One possibility is that the efficacy of selection (*N_e_s*) against deleterious mutations may vary across classes of mitochondrial-encoded factors and across lineages with different *N_e_*, resulting in differential accumulation of deleterious substitutions in mtDNA to drive compensatory molecular evolution. Although previous work has documented higher deleterious substitution rates in mt-tRNAs relative to nuclear tRNAs (Lynch 1996, 1997), estimating the fitness effects of RNA nucleotide substitutions is notoriously difficult, and high levels of sequence similarity and incomplete assemblies and annotations often confound distinguishing between cyt-tRNA orthologs and paralogs (Rogers et al. 2010). New studies are needed to estimate the number of nuclear tRNA copies and the fraction that are actively transcribed and functional, as nuclear tRNA paralogs and DNA fragments translocated from the mtDNA to the nuclear genome (i.e., NUMTs) would likely have been conflated during the initial investigations of tRNA evolution nearly 20 years ago. Our analyses warrant new investigation of mitochondrial versus nuclear tRNA evolution to provide better understanding of the relative contributions of compensatory evolution and constraint to the patterns of substitution observed in proteins interacting with mitochondrial versus cytoplasmic factors.

## Materials and Methods

### Sequences and Alignments

From Ensembl v80, we obtained the longest protein-coding transcript for human aaRS genes for which we could confidently exclude the presence of paralogs and which did not have a dual role in aminoacylating both mitochondrial and cytosolic tRNAs (Lo et al. 2014). All other mammal sequences were obtained by identifying the longest protein-coding transcript of high-confidence, 1:1 orthologs of human aaRS genes, excluding transcripts with synonymous site saturation (pairwise *d*_S_ with humans >1) from Ensembl v80. All bird and *D. melanogaster* orthologous transcripts were similarly obtained, without regard to pairwise distance with humans. Known orthologs for the remaining four *Drosophila* species were retrieved from FlyBase (FlyBase Consortium 2003). We excluded all orthologs with lengths shorter than 70% of the mean length for all other orthologs in the alignment.

Proteins targeted to the mitochondria contain a rapidly evolving N-terminal mitochondrial-targeting leader peptide, and these sequences have the potential to confound the comparative analysis of the evolutionary rates of these proteins. We identified mitochondrial-targeting cleavage sites using TargetP v1.1 (Nielsen et al. 1997; Emanuelsson et al. 2000) and manually cleaved predicted targeting sequences from all mt-aaRS transcripts. We aligned all sequences using MUSCLE v3.8 (Edgar 2004) and systematically removed regions of poor alignment using Gblocks v0.91b (Castresana 2000) using the options: -t=c −b1 = “$b1” −b2 = “$b1” -b3 = 1 -b4 = 6 -b5 = h, where b1 = 70% of sampled sequences. All transcript accessions, sequence alignments, and gene trees (see below) have been deposited in Dryad (http://dx.doi.org/10.5061/dryad.4p24g).

### Maximum Likelihood Codon Analysis

We used codeml (model = 0, NSsites = 0) in PAML v4.4 (Yang 2007) to estimate a single value of *ω* for each aaRS in order to contrast substitution rates between cyt-aaRS and mt-aaRS. To account for phylogenetic discordance among aaRS genes, which occurred rarely in birds and flies but for every aaRS gene in mammals, we used gene trees inferred by RAxML (Stamatakis 2006) with options: -m GTRGAMMA -p 12345. To test for sites under positive selection we used a likelihood ratio test of significance (2Δλ, where Δλ equals the difference in log likelihood scores between M2a and M1a) using the χ^2^ distribution and two degrees of freedom (Yang 2007). Site model M1a estimates *ω* for two classes of sites that are either evolving under purifying selection (*ω*_0_ < 1) or accumulating substitutions with no selection (*ω*_1_ = 1), whereas M2a estimates *ω* for three classes of sites: those evolving under purifying selection (*ω*_0_ < 1), no selection (*ω*_1_ = 1), or under positive selection (*ω*_2_ > 1). Additionally, we used BEB (Yang et al. 2005) to calculate the posterior probability that any site was evolving under positive selection.

### Population Data Analysis

Counts of *P_n_*, *P_s_*, *D_n_*, and *D_s_* for aaRS genes in *D. melanogaster* were obtained from the *Drosophila* Population Genomics Project (Langley et al. 2012) and for humans were from Bustamante et al. (2005). We used the software package DoFE v3.0 (http://www.lifesci.sussex.ac.uk/home/Adam_Eyre-Walker/Website/Software.html, last accessed June 19, 2015) to estimate ${\text{NI}}_{TG}=\frac{\Sigma \;D_{si}P_{ni}/(P_{si}+D_{si})}{\Sigma \;P_{si}D_{ni}/(P_{si}+D_{si})}$ (Stoletzki and Eyre-Walker 2011) with 95% confidence intervals from 1,000 bootstrap samples, although we did not detect significant heterogeneity among individual aaRS gene contingency tables in either humans or *D. melanogaster* (*P* > 0.05; Woolf’s test). Fisher’s exact tests of the two-by-two MK contingency tables of polymorphic and fixed sites were performed in R and a Bonferroni correction was used to account for multiple tests within each class of aaRS genes in *D. melanogaster* and humans.

### Gene Expression Analysis

We obtained raw RNA-seq data sets from the NCBI Sequence Read Archive and reference genomes and gene annotations from the Illumina iGenomes database (supplementary table S2, Supplementary Material online). We trimmed adapters and low quality bases from both 5′- and 3′-ends until the minimum aggregate quality score (Q_sanger_) was ≥ 20.0. We mapped trimmed reads to a reference genome using TopHat v2.0.7 (Trapnell et al. 2009) and used reference gene annotations to provide intron–exon junctions. We used Cufflinks v2.2.0 (Trapnell et al. 2010; Roberts, Pimentel, et al. 2011) to perform a reference-guided transcript assembly and to estimate relative transcript abundance, correcting for biases in the nonuniformity of transcript distributions (Roberts, Trapnell, et al. 2011). We estimated gene expression as mapped fragments per kilobase of transcript per million transcripts mapped (FPKM), which is conceptually similar to the commonly used reads per kilobase per million reads sequenced (Trapnell et al. 2010). We used Cuffmerge and Cuffdiff v2.2.0 (Trapnell et al. 2010) to merge the annotations from replicate data sets and to calculate FPKM for each gene in each tissue type and life stage. We used a quartile library normalization method to improve the accuracy of expression calls for low abundance transcripts (Bullard et al. 2010) and to scale among replicate libraries.

### Statistical Analyses

We used a GLM to fit the regression of substitution rates (*ω*, *d*_N_, and *d*_S_ estimated from our codeml analyses (model = 0) of mammals, birds, and *Drosophila*) on the log-transformed values of FPKM for a given tissue. We tested for a significant difference in the residuals of fit for the combined pool of all aaRS genes on FPKM using a Mann–Whitney *U* test. We calculated AIC for the regression of *d*_N_/*d*_S_ on FPKM (Expression), on mt-aaRS/cyt-aaRS (Class), on Class + Expression, and on Class × Expression. We calculated Akaike weights (w_i_) and the change in AIC model (ΔAIC) using the qpcR package (Spiess and Ritz 2011) in R. All statistical analysis was performed in R (R Development Core Team 2011).

## Data Accessibility

All transcript accessions, sequence alignments, and gene trees have been deposited in Dryad (http://dx.doi.org/10.5061/dryad.4p24g). 

## Supplementary Material

Supplementary figures S1–S4 and tables S1 and S2 are available at *Molecular Biology and Evolution* online (http://www.mbe.oxfordjournals.org/).

Supplementary Data
