# Supplementary material for: The Roles of Compensatory Evolution and Constraint in Aminoacyl tRNA Synthetase Evolution
Source: Mol Biol Evol. 2015 Sep 28;33(1):152–61. doi: 10.1093/molbev/msv206 (PMC4693975; doi:10.1093/molbev/msv206)
Supplement: Supplementary Data [file supp_33_1_152__index.html]

The Roles of Compensatory Evolution and Constraint in Aminoacyl tRNA Synthetase Evolution — The Roles of Compensatory Evolution and Constraint in Aminoacyl tRNA Synthetase Evolution — The Roles of Compensatory Evolution and Constraint in Aminoacyl tRNA Synthetase Evolution — Supplementary Data 

# The Roles of Compensatory Evolution and Constraint in Aminoacyl tRNA Synthetase Evolution

## Supplementary Data

files

- Supplementary Data - pdf file
