# Supplementary material for: The Roles of Compensatory Evolution and Constraint in Aminoacyl tRNA Synthetase Evolution
Source: Mol Biol Evol. 2015 Sep 28;33(1):152–61. doi: 10.1093/molbev/msv206 (PMC4693975; doi:10.1093/molbev/msv206)
Supplement: Supplementary Data [file supp_msv206_aaRS_supplemental_materials_submission.pdf]

Supplemental Figure 1

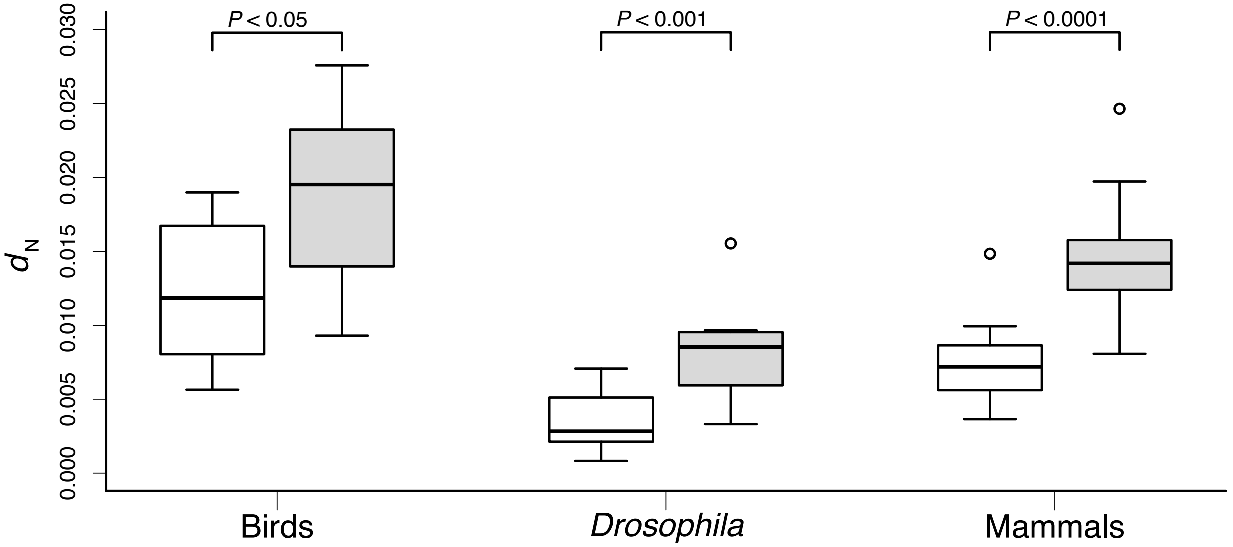

Supplemental Figure 1: Nonsynonymous substitution rates of cyt-aaRS (white boxes) and mt-aaRS (gray boxes). Per-gene estimates of the average nonsynonymous substitution rate (d_N) per-branch were generated using codeml model 0 in PAML. *P* values indicate significant differences based on Mann-Whitney *U* tests.

Supplemental Figure 2

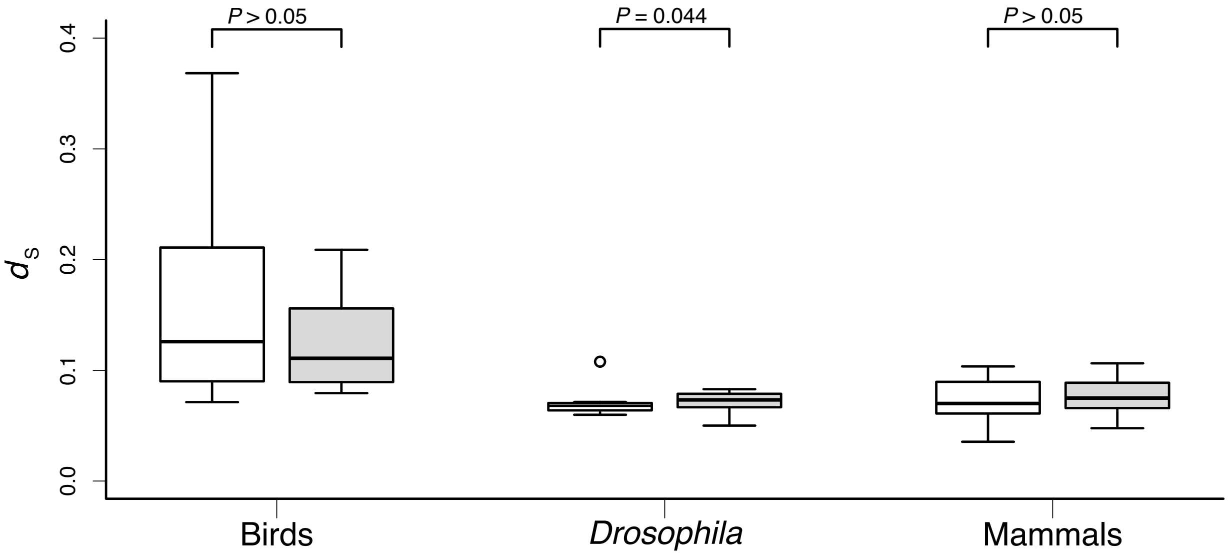

Supplemental Figure 2: Synonymous substitution rates of cyt-aaRS (white boxes) and mt-aaRS (gray boxes). Per-gene estimates of the average synonymous substitution rate (d_S) per-branch were generated using codeml model 0 in PAML. *P* values indicate significant differences based on Mann-Whitney *U* tests.

Supplemental Figure 3

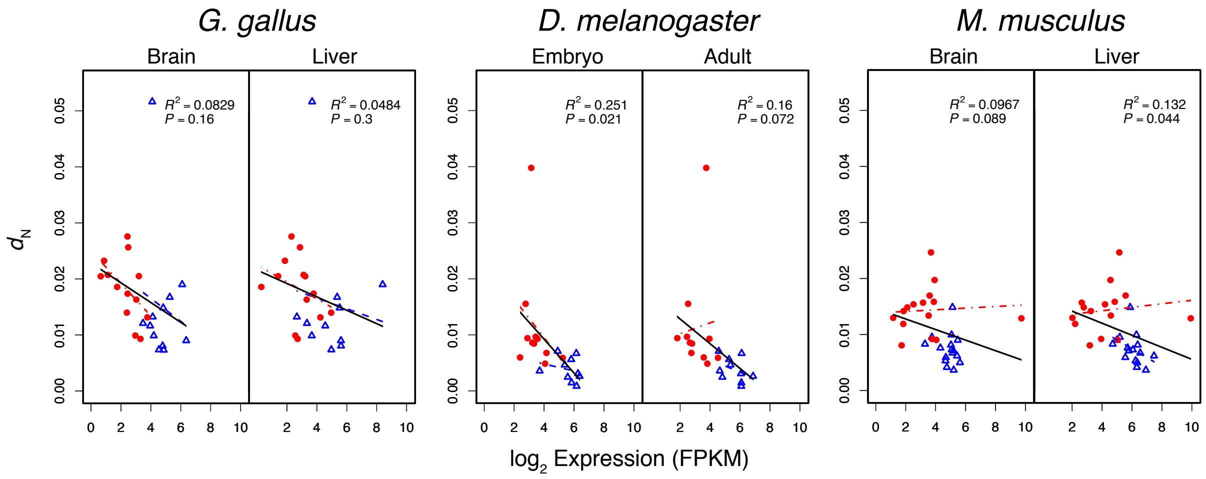

Supplemental Figure 3: Relationship between transcript levels (FPKM) and d_N. GLM regressions are shown separately for mt-aaRS (closed circles, red dashed line) and cyt-aaRS (open triangles, blue dashed line) and for all aaRS genes (solid black line). R^2 and P-values are from regressions using data from all aaRS genes.

Supplemental Figure 4

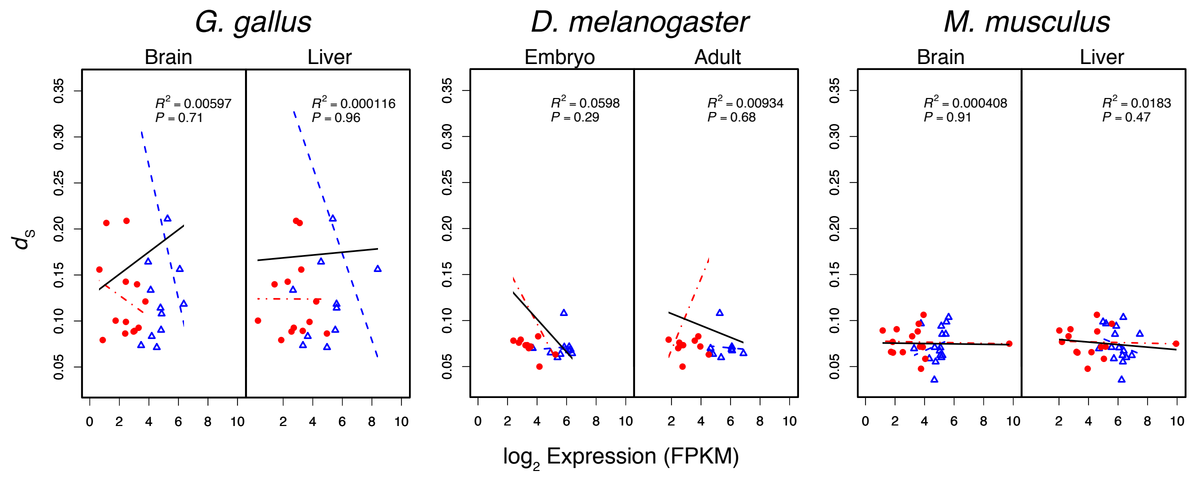

Supplemental Figure 4: Relationship between transcript levels (FPKM) and d_S. GLM regressions are shown separately for mt-aaRS (closed circles, red dashed line) and cyt-aaRS (open triangles, blue dashed line) and for all aaRS genes (solid black line). R^2 and P-values are from regressions using data from all aaRS genes.

Supplemental Table 1. Results from AIC model comparisons.

Species	Tissue/Stage	Model[1]	AIC[2]	DAIC[3]	w_i[4]
G. gallus	Brain	**Expression**	-79.774	0.000	0.472
		Expression + Class	-78.288	1.486	0.225
		Class	-77.968	1.806	0.191
		Expression * Class	-76.891	2.883	0.112
	Liver	**Class**	-74.107	0.000	0.489
		Expression + Class	-72.631	1.476	0.234
		Expression * Class	-71.802	2.305	0.155
		Expression	-71.329	2.779	0.122
D. melanogaster	Embryo	**Class**	-76.012	0.000	0.386
		Expression + Class	-75.245	0.766	0.263
		Expression	-75.173	0.838	0.254
		Expression * Class	-73.248	2.764	0.097
	Adult	**Class**	-94.979	0.000	0.461
		Expression + Class	-93.971	1.008	0.278
		Expression * Class	-93.763	1.216	0.251
		Expression	-87.248	7.731	0.010
M. musculus	Brain	**Class**	-101.512	0.000	0.626
		Expression + Class	-99.526	1.985	0.232
		Expression * Class	-98.545	2.967	0.142
		Expression	-83.652	17.860	8.285e-05
	Liver	**Class**	-101.512	0.000	0.602
		Expression + Class	-99.770	1.742	0.252
		Expression * Class	-98.675	2.837	0.146
		Expression	-83.842	17.670	8.764e-05

[1]Best model shown in bold text
[2]Best model has lowest raw AIC value
[3]Change in raw AIC value from best model
[4]Akaike weights can be thought of as the probability that the given model is the best model

Supplemental Table 2. Gene expression datasets

Species	Tissue	Stage	Sequencing Platform	Library layout	Experiment	Accession	Reference Genome
M. musculus	Whole Brain	E14.5	Illumina HiSeq 2000	Paired	SRX186051	SRR567498	USSC mm10
M. musculus	Whole Brain	E14.5	Illumina HiSeq 2000	Paired	SRX186051	SRR567499	USSC mm10
M. musculus	Liver	E14	Illumina HiSeq 2000	Paired	SRX186053	SRR567502	USSC mm10
M. musculus	Liver	E14	Illumina HiSeq 2000	Paired	SRX186053	SRR567503	USSC mm10
G. gallus	Hypothalamus	Adult	Illumina HiSeq 2000	Paired	SRX316899	SRR924551	WASHUC2
G. gallus	Liver	Adult	Illumina HiSeq 2000	Paired	SRX316903	SRR924555	WASHUC2
D. melanogaster	Whole Fly	E12-16	Illumina Genome Analyzer	Single	SRX012974	SRR030226	NCBI build5.3
D. melanogaster	Whole Fly	E12-16	Illumina Genome Analyzer	Single	SRX012974	SRR030227	NCBI build5.3
D. melanogaster	Whole Fly	Adult	Illumina Genome Analyzer	Single	SRX012976	SRR030230	NCBI build5.3
D. melanogaster	Whole Fly	Adult	Illumina Genome Analyzer	Single	SRX012976	SRR030231	NCBI build5.3
